# Clinical and Microbiological Characteristics of Recurrent Escherichia coli Bacteremia

**DOI:** 10.1128/Spectrum.01399-21

**Published:** 2021-12-08

**Authors:** Tatsuya Kobayashi, Mahoko Ikeda, Yuta Okada, Yoshimi Higurashi, Shu Okugawa, Kyoji Moriya

**Affiliations:** a Department of Infectious Diseases, The University of Tokyo Hospital, Tokyo, Japan; b Department of Infection Control and Prevention, The University of Tokyo Hospital, Tokyo, Japan; The National University of Singapore and the Genome Institute of Singapore

**Keywords:** recurrent *Escherichia coli* bacteremia, enterobacterial repetitive intergenic consensus polymerase chain reaction, genetic identity, virulence factor

## Abstract

The causative agents of recurrent Escherichia coli bacteremia can be genetically identical or discordant, but the differences between them remain unclear. This study aimed to explore these differences, with regard to their clinical and microbiological features. Patients were recruited from a Japanese tertiary teaching hospital based on blood culture data and the incidence of recurrent E. coli bacteremia. We compared the patients' clinical and microbiological characteristics between the two groups (those with identical or discordant E. coli bacteremia) divided by the result of enterobacterial repetitive intergenic consensus PCR. Among 70 pairs of recurrent E. coli bacteremia strains, 49 pairs (70%) were genetically identical. Patients with genetically identical or discordant E. coli bacteremia were more likely to have renal failure or neoplasms, respectively. The virulence factor (VF) scores of genetically identical E. coli strains were significantly higher than those of genetically discordant strains, with the prevalence of eight VF genes being significantly higher in genetically identical E. coli strains. No significant differences were found between the two groups regarding antimicrobial susceptibility and biofilm formation potential. This study showed that genetically identical E. coli bacteremia strains have more VF genes than genetically discordant strains in recurrent E. coli bacteremia.

**IMPORTANCE** Escherichia coli causes bloodstream infection, although not all strains are pathogenic to humans. In some cases, this infection reoccurs, and several reports have described the clinical characteristics and/or molecular microbiology of recurrent Escherichia coli bacteremia. However, these studies focused on patients with specific characteristics, and they included cases caused by microorganisms other than Escherichia coli. Hence, little is known about the pathogenicity of Escherichia coli isolated from the recurrent one. The significance of our study is in evaluating the largest cohorts to date, as no cohort studies have been conducted on this topic.

## INTRODUCTION

Escherichia coli is the most frequent pathogen causing bloodstream infections in many situations (1–4). However, not all E. coli strains are pathogenic to humans, and commensal E. coli represents a colonizer of the human gastrointestinal microbiota (5). The majority of pathogenic E. coli strains belong to the phylogenetic group B2 (6). Conversely, groups A and B1 account for a larger proportion of commensal strains (7).

E. coli has various virulence factors (VFs), including toxins, adhesins, siderophores, and polysaccharide capsules (8, 9). Numerous VFs are related to the pathogenicity of E. coli, with a wide range of pathogenic activities (10). In addition to VFs, antimicrobial resistance and biofilm formation also make it difficult to eliminate microorganisms from the human body (11, 12).

Two or more E. coli bacteremia episodes occur in 3–28% of all cases (13–20), although the prevalence of recurrent E. coli bacteremia depends on the situation and its definition. Several reports have described the clinical characteristics and/or molecular microbiology of recurrent E. coli bacteremia (13, 15, 18–21). However, these studies are case series, studies focused on patients with specific characteristics, such as hematological malignancy, or studies of bacteremia caused by microorganisms other than E. coli. Moreover, little is known about the pathogenicity of E. coli isolated from recurrent E. coli bacteremia.

The aim of this study was to identify the differences in clinical and microbiological characteristics of E. coli bacteremia depending on genetic identity. To our knowledge, this study is the largest cohort ever conducted solely on patients with recurrent E. coli bacteremia.

## RESULTS

### Inclusion of the study participants and differentiation by using ERIC-PCR.

**FIG 1:**
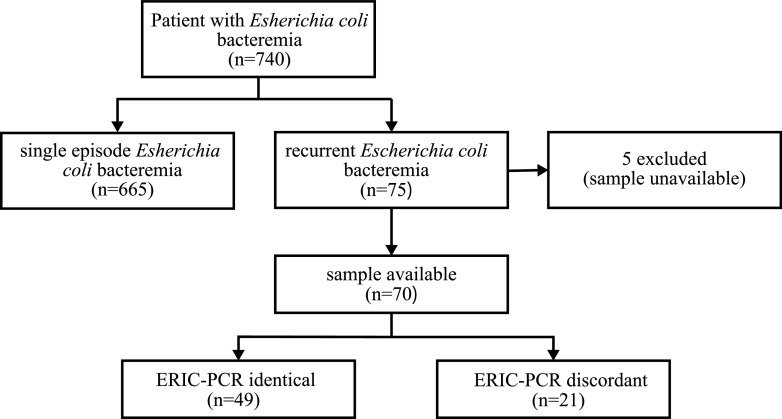
Flow chart of the classification of study participants according to the ERIC-PCR results. ERIC-PCR, enterobacterial repetitive intergenic consensus PCR.

In total, 740 patients with bloodstream infections caused by E. coli were identified between April 2013 and March 2019. Among them, 75 patients (10.1%) had a second episode of E. coli bacteremia. Five patients were excluded because their culture samples were unavailable. ERIC-PCR was performed by using 70 pairs of E. coli isolates from the remaining 70 patients. Among the 70 pairs of E. coli isolates, 49 pairs (70%) were genetically identical (Fig. 1).

### Comparison of patients with genetically identical and genetically discordant E. coli bacteremia.

The demographic and clinical characteristics of patients in the two groups are listed in Table 1. Most of the clinical characteristics were not significantly different between the two groups. Compared with patients with genetically discordant E. coli bacteremia, those with genetically identical E. coli bacteremia were significantly more likely to have renal failure (*P* = 0.0079). In contrast, patients with genetically discordant E. coli bacteremia were more likely to have neoplasms (*P* = 0.038).

**TABLE 1:** Clinical characteristics of patients with recurrent E. coli bacteremia^*a*^

Characteristics	ERIC-PCR identical strains (*n* = 49)	ERIC-PCR discordant strains (*n* = 21)	*P*-value
Demographics			
Median age (IQR)	72 (63–78.5)	75 (68–80)	0.175
Male-no. (%)	29 (59.2)	11 (52.3)	0.61
Acquisition-no. (%)			0.387
Hospital-acquired	16 (32.7)	4 (19.0)	
Community-acquired	33 (67.3)	17 (81.0)	
Underlying disease/condition-no. (%)			
Diabetes mellitus	21 (42.9)	14 (66.7)	0.117
Neoplasm	21 (42.9)	15 (71.4)	0.038
Immunosuppressant use	20 (40.8)	4 (19.1)	0.103
Transplantation	5 (10.2)	1 (4.8)	0.661
Renal failure	17 (34.7)	1 (4.8)	0.0079
Surgery within 30 days	2 (4.1)	0 (0.0)	1
Foreign body	14 (28.6)	5 (23.8)	0.776
Chemotherapy	10 (20.4)	6 (28.6)	0.538
Source of bacteremia-no. (%)			0.072
Biliary tract	13 (26.5)	12 (57.1)	
Urinary	19 (38.8)	3 (14.3)	
Others	16 (32.7)	7 (33.3)	
Pitt bacteremia score ≧4-no. (%)	2 (4.1)	0 (0.0)	1
Relapse within 60 days-no. (%)	20 (40.8)	7 (33.3)	0.603
Polymicrobial bacteremia-no. (%)	4 (8.2)	3 (14.3)	0.421

a IQR, interquartile range; ERIC-PCR, enterobacterial repetitive intergenic consensus PCR.

### Distribution of phylogenetic groups and sequence types among recurrent E. coli bacteremia isolates.

As shown in Tables 2 and 3, there were significant differences between genetically identical strains and discordant strains in the distribution of phylogenetic groups (*P* = 0.0001) and sequence types (STs) (*P* = 0.0063). In both groups, that with the largest number of strains was categorized as the phylogenetic group B2. The percentages of phylogenetic groups B2, ST 131, ST 73, and ST 1193 were higher in genetically identical strains than in genetically discordant strains.

**TABLE 2:** Distribution of phylogenetic groups among recurrent E. coli bacteremia isolates

Phylogenetic group	ERIC-PCR identical strains (*n* = 49) (no. [%])^*a*^	ERIC-PCR discordant strains (*n* = 21) (no. [%])	*P*-value
A	0 (0.0)	1 (4.8)	0.0001
B1	0 (0.0)*	6 (28.6)*	
B2	43 (87.8)*	10 (47.6)*	
C	0 (0.0)	0 (0.0)	
D	0 (0.0)	0 (0.0)	
E	4 (8.2)	2 (9.5)	
F	2 (4.1)	2 (9.5)	

a ERIC-PCR, enterobacterial repetitive intergenic consensus PCR.

*Significant, *P* = 0.002 (Fisher's exact test with the Bonferroni correction).

**TABLE 3:** Distribution of STs among recurrent E. coli bacteremia isolates

Sequence type	ERIC-PCR identical strains (*n* = 49) (no. [%])^*a*^	ERIC-PCR discordant strains (*n* = 21) (no. [%])	*P*-value
ST131	18 (36.7)	3 (14.2)	0.0063
ST95	9 (18.4)	4 (19.1)	
ST73	7 (14.3)	0 (0.0)	
ST1193	4 (8.2)	0 (0.0)	
ST357	1 (2.0)	1 (4.8)	
Others	10 (20.4)	13 (61.9)	

a ERIC-PCR, enterobacterial repetitive intergenic consensus PCR.

In *post hoc* analyses performed to determine which groups differed, the distribution of STs was not significantly different for all combinations. The phylogenetic group B2 was significantly more common than phylogenetic group B1 in the genetically identical strains (Bonferroni-corrected *P* = 0.002).

### Distribution of VFs.

**FIG 2:**
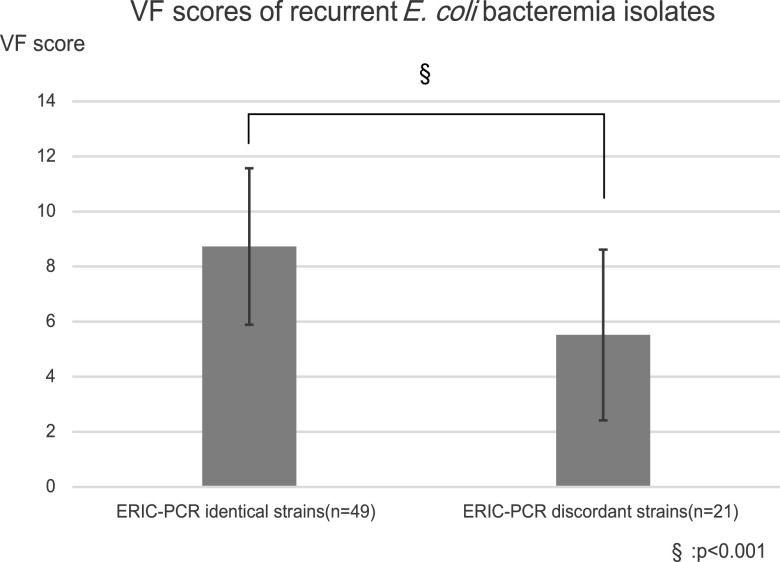
Differences in the proportion of VF genes between ERIC-PCR identical strains and genetically discordant strains. ERIC-PCR identical strains have significantly more VF genes than genetically discordant strains. ERIC-PCR, enterobacterial repetitive intergenic consensus PCR; VF, virulence factor.

The difference in the distribution of 20 VFs between genetically identical and discordant strains is provided in Table 4. Of the 20 VFs evaluated, the prevalence rates of eight VF genes (*sfaD/E* [*P* = 0.049], *iha* [*P* = 0.0095], *fyuA* [*P* = 0.0019], *cnf1* [*P* = 0.027], *sat* [*P* = 0.017], *hlyA* [*P* = 0.027], *KpsMT2* [*P* = 0.041], and *usp* [*P* = 0.002]) were significantly higher in genetically identical E. coli strains than in the discordant strains. In addition, VF scores of genetically identical E. coli strains were significantly higher than those of genetically discordant strains (*P* = 0.0003) (Fig. 2).

**TABLE 4:** Distribution of virulence factor (VF) genes among recurrent E. coli bacteremia isolates

VF genes	ERIC-PCR identical strains (*n* = 49) (no. [%])^*a*^	ERIC-PCR discordant strains (*n* = 21) (no. [%])	*P*-value
*afaB/C*	3 (6.1)	1 (4.8)	1
*fimH*	49 (100.0)	20 (95.2)	0.3
*sfaD/E*	9 (18.4)	0 (0.0)	0.049
*papC*	21 (42.9)	4 (19.0)	0.064
*papG2*	14 (28.6)	3 (14.3)	0.24
*iha*	26 (53.1)	4 (19.0)	0.0095
*iucD*	29 (59.2)	8 (38.1)	0.124
*iutA*	29 (59.2)	8 (38.1)	0.124
*fyuA*	46 (93.9)	13 (61.9)	0.0019
*iroN*	13 (26.5)	4 (19.0)	0.561
*cnf1*	11 (22.4)	0 (0.0)	0.027
*sat*	25 (51.0)	4 (19.0)	0.017
*hlyA*	11 (22.4)	0 (0.0)	0.027
*KpsMT2*	39 (79.6)	11 (52.4)	0.041
*cvaC*	3 (6.1)	4 (19.0)	0.186
*ibeA*	10 (20.4)	3 (14.3)	0.741
*ompT*	3 (6.1)	4 (19.0)	0.186
*TcpC*	8 (16.3)	2 (9.5)	0.712
*usp*	42 (85.7)	10 (47.6)	0.002
*traT*	38 (77.6)	13 (61.9)	0.242

a ERIC-PCR, enterobacterial repetitive intergenic consensus PCR.

### Possession of ESBL genes/antimicrobial susceptibilities.

Among 70 E. coli bacteremia isolates that caused the first bacteremia episode, 13 strains (18.6%) carried ESBL genes. Of those 13 ESBL-producing E. coli strains, 10 belonged to CTX M-9 group and three to CTX M-1 group. All isolates that were positive in the ESBL phenotypic test carried ESBL genes.

As shown in Table 5, no significant differences in antimicrobial susceptibilities including ESBL production were found between the two groups.

**TABLE 5:** Antimicrobial susceptibilities among recurrent E. coli bacteremia isolates^*a*^

Antimicrobial susceptibilities	ERIC-PCR identical strains (*n* = 49) (no. [%])	ERIC-PCR discordant strains (*n* = 21) (no. [%])	*P*-value
ESBL production	11 (22.4)	2 (9.5)	0.317
Quinolone resistance	22 (44.9)	4 (19.1)	0.059
Ampicillin resistance	28 (57.1)	8 (38.1)	0.194

a ERIC-PCR, enterobacterial repetitive intergenic consensus PCR; ESBL, extended-spectrum β-lactamase.

### Biofilm formation ability.

Compared with genetically discordant strains, there were no significant differences in the biofilm formation abilities of genetically identical strains (Table 6).

**TABLE 6:** Biofilm formation ability among recurrent E. coli bacteremia isolates^*a*^

Biofilm formation	ERIC-PCR identical strains (*n* = 49) (no. [%])	ERIC-PCR discordant strains (*n* = 21) (no. [%])	*P*-value
Biofilm formation (LB Lennox broth)	21 (42.9)	6 (28.6)	0.296
Biofilm formation (BHI broth)	17 (34.7)	8 (38.1)	0.792

a ERIC-PCR, enterobacterial repetitive intergenic consensus PCR; BHI, brain heart infusion.

## DISCUSSION

To our knowledge, among comparative studies limited to recurrent E. coli bacteremia classified by genetic homology, this study evaluated the largest cohorts to date. The prevalence of recurrent E. coli bacteremia in this study was 10.1%, which is within the range reported in previous studies (13–20). However, the prevalence of recurrent E. coli bacteremia varies across the studies (13–20). This variation is presumably influenced by the definition of recurrence, study population, and study design.

In previous studies that categorized recurrent E. coli bacteremia by genetic identity, E. coli strains were genetically characterized using pulsed-field gel electrophoresis (PFGE) (15, 20), repetitive extragenic palindromic sequence PCR (13), and ribotyping (15, 21). Compared with other PCR-based typing techniques, PFGE is a powerful and accurate technique for strain typing, but it is laborious because of its low throughput (22). In a large sample size study, Casarez et al. reported that ERIC-PCR was similarly effective to PFGE in the differentiation of E. coli strains (23). With the advent of next-generation genome sequencing techniques, whole-genome sequencing (WGS) provides higher discrimination power for typing of pathogens including *E coli* compared to PCR-based typing techniques (24, 25). To the best of our knowledge, there are no studies that directly compare the performance of WGS to that of ERIC-PCR, so the quantitative difference in resolution between these two methods is unknown. A study of recurrent Staphylococcus aureus bacteremia showed that the amount of SNP variation by WGS is consistent with the PFGE, and authors concluded that no novel insights were provided by WGS in the study (26). Although the result may not be applicable to our study because of the difference in the species of bacteria studied, as for repeated bacteremia in a single patient, the result of WGS may be consistent with that of PCR-based typing techniques, which has lower discrimination power compared to WGS. This consistency may well be the case in repeated infections due to the colonization of the same bacteria. WGS technique has changed the landscape of genomic science; however, its high costs and the need for bioinformatics analysis can contribute to delays of its replacement of PCR-based typing techniques (27). Considering the above, we used ERIC-PCR to classify the genetic identity of recurrent E. coli bacteremia in this study, and we found that 70% of the recurrent E. coli bacteremia cases were caused by genetically identical strains. This percentage was similar to that reported for ESBL-producing E. coli and Klebsiella pneumoniae (67.8%) (20) but was higher than that reported in a previous study on E. coli (47.7%) (13).

The comparison between genetically identical strains and genetically discordant strains showed neoplasms as a factor that correlated with genetic discordance. In contrast, patients with genetically identical E. coli bacteremia were significantly more likely to have renal failure. In a previous study of recurrent bacteremia caused by ESBL-producing E. coli and Klebsiella pneumoniae, there was no significant difference in comorbidity, including neoplasms and renal failure, between the two groups based on genetic identity (20). Although it is difficult to interpret the results of our study because there are no similar studies referring to these comorbidities on E. coli alone, genetically identical E. coli may have not been eliminated from the patients during the treatment of the prior bacteremia episodes. Zerr et al. indicated that *Enterobacteriales* strains of the same STs and resistance genotypes can persistently colonize the intestinal tract even after treatment with effective antimicrobial agents, which may lead to recurrent infections (28).

Among the E. coli strains included in this study, phylogenetic group B2 strains accounted for the largest percentage in both groups. However, the proportion of phylogenetic group B2 strains was significantly higher in the genetically identical group. Given that phylogenetic group B2 strains are more likely to cause infections than other strains (6), this result is logical. As for STs, the percentages of ST 131, ST 73, and ST 1193, which belong to phylogenetic group B2, were higher in the genetically identical group. However, the proportion of the ST 95 strain, which also belongs to phylogenetic group B2, was almost the same between the two groups. Thus, not all phylogenetic B2 strains may be associated with genetic identity. The results of the *post hoc* analysis did not reveal any differences in the combinations of STs between the two groups. The lack of significant differences in the STs between groups may be partly due to limited statistical power due to the small sample size. ST 131 is one of the main ESBL-producing E. coli clones isolated worldwide (29); however, there were no significant differences in antimicrobial susceptibility between the two groups, including ESBL production.

Among the eight VF genes that showed significant differences, *cnf1*, *hlyA*, and *sat* encode toxins; *sfaD/E* and *iha* regulate adhesins; *fyuA* regulates siderophores; *KpsMT2* and *usp* encode capsules and uropathogenic-specific proteins, respectively. Previous studies that analyzed recurrent urinary tract infection (UTI) in women have showed the presence of numerous VF genes that regulate various factors, including toxins, adhesins, iron-acquisition systems, capsules, fimbriae, and other factors in relation to recurrent E. coli UTI (30–33). In our study, genetically identical strains possessed significantly more VFs than genetically discordant strains. There was no statistically significant difference, but there was a trend toward more UTIs in the genetically identical group. According to a previous report, E. coli bacteremia strains originating from urinary tract infective foci harbored more VF genes than those from non-urinary tract infective foci (34). Furthermore, the percentages of ST 131, ST 73, and ST 1193 were higher in the genetically identical group, as aforementioned. There are some reports indicating that these strains carry prototypic VF genes (35, 36). Thus, the high prevalence of UTIs and several STs in the genetically identical group may be one of the reasons for the higher VF score. However, making comparisons of these studies is difficult because their selection of VF genes differs from each other. Moreover, only a few reports of recurrent E. coli bacteremia have described the distribution of VF genes; therefore, further investigation is required to clarify the distribution of VF genes in recurrent E. coli bacteremia and how VFs contribute to recurrence. The roles of VFs are diverse, suggesting the involvement of multiple VFs in the establishment of recurrent infections.

Several VFs have been reported to be related to biofilm formation in ESBL E. coli strains (37). Biofilms are a layer of bacteria attached to biological tissues or artificial device surfaces, and biofilms have been reported to have several advantages for the survival of bacteria: increased capacity of bacterial conjugation (38), increased interspecific metabolic cooperation (39), and increased needs for higher concentrations of antibiotics (40). A study focused on E. coli associated with recurrent cystitis showed that recurrent infection isolates had better biofilm formation capability than single infection isolates (41), although this difference was not significant.

This study has several limitations. First, our study was a hospital-based, retrospective, single-center study. The description of E. coli strains identified in this study might not be entirely generalizable to E. coli strains collected in different situations. Second, we analyzed only in-hospital data, and the sample size was too small to perform multivariate analyses. For *post hoc* multiple comparison analyses, the small sample size limited the statistical power to detect significant differences. Third, relapse and reinfection were not distinguished in this study, both of which can be included in genetically identical strains group. To exclude cases of relapse to the extent possible, we selected cases in which the interval between the first and second positive blood cultures was more than 4 weeks. Fourth, WGS was not performed as mentioned above, which leads to the reduction of discrimination power regarding genetic identity. Despite these limitations, this study can help identify the clinical and microbiological characteristics that predispose patients to recurrent E. coli bacteremia.

In conclusion, among recurrent E. coli bacteremia strains, genetically identical strains have significantly more VF genes than genetically discordant strains. We have also shown the clinical characteristics of patients with recurrent E. coli bacteremia. Further research is required to explore how VFs may contribute to E. coli bacteremia recurrence.

## MATERIALS AND METHODS

### Study design and patients.

This retrospective study was conducted at the University of Tokyo Hospital, a 1,217-bed tertiary teaching hospital in Japan. Patients with E. coli bacteremia were selected from a database of blood culture results between April 2013 and March 2019. Patients with recurrent E. coli bacteremia were selected from these patients. Information on demographics, underlying diseases/conditions, site of acquisition, source of bacteremia, Pitt bacteremia score (42) calculated on the day of positive blood culture, time between first and second episode, and presence or absence of polymicrobial infection was extracted from the medical records. Recurrent E. coli bacteremia strains were categorized into two groups based on genetic identity. As for microbiological characteristics, we analyzed E. coli strains that caused the first bacteremia episode. The study protocol was approved by the ethics committee of the Graduate School of Medicine and Faculty of Medicine, University of Tokyo (approval number 10799). The requirement for written informed consent was waived because of the observational, retrospective design of the study.

### Classification by genetic identity using ERIC PCR.

Enterobacterial repetitive intergenic consensus PCR (ERIC-PCR) analysis was performed using primers ERIC-1 and ERIC-2R, as described previously (43). PCR was performed using EmeraldAmp MAX PCR Master Mix (TaKaRa Bio Inc., Shiga, Japan). The results of ERIC-PCR were compared using GelJ v2.0, a software tool for analyzing DNA fingerprint gel images (44). Isolates with more than 90% identical profiles were defined as genetically identical pairs.

### Definitions.

Recurrent E. coli bacteremia was defined as a second episode of E. coli bacteremia occurring at least 4 weeks from the date of the positive blood culture of the first E. coli bacteremia episode. All patients' first episodes of bacteremia were appropriately treated prior to the second episode. E. coli bacteremia was categorized as community-acquired or nosocomial. Nosocomial E. coli bacteremia was defined as that which occurred at 48 h or more after hospital admission, and community-acquired E. coli bacteremia was defined as anything other than nosocomial E. coli bacteremia.

The diagnosis of UTI was based on the detection of E. coli that has grown in the quantity of 10^5 CFU per milliliter in the urine culture, with clinical symptoms, such as fever (≥37.5°C), pain during urination, or costovertebral angle tenderness.

Biliary tract infection was defined based on the Tokyo guidelines 2018 (45). A definite diagnosis of cholangitis was made when all the following were positive: i) systemic inflammation signs, such as fever and/or shaking chills, elevation of inflammation sign on blood test; ii) signs of cholestasis, such as jaundice, abnormal liver function, and biliary function tests; and ii) biliary dilatation or evidence of the etiology on imaging. Definite cholecystitis was defined as the patient meeting all the following criteria: i) localized clinical signs, such as Murphy’s sign and pain or tenderness in the right upper quadrant; ii) systemic inflammation signs, such as fever, elevation of C-reactive protein level or white blood cell count; and iii) imaging findings and characteristic of acute cholecystitis.

Renal failure was defined as having a serum creatinine level ≥1.5 mg/dl or requirement of dialysis therapy.

### Identification of E. coli and antimicrobial susceptibility testing.

Blood samples were cultured using the BacT/Alert 3D Microbial Detection System (bioMérieux, Inc., Durham, NC).

All E. coli isolates were identified using the MicroScan WalkAway system (Beckman Coulter, Tokyo, Japan) or matrix-assisted laser desorption/ionization time-of-flight mass spectrometry (MALDI Biotyper; Bruker Daltonics, Germany). Antimicrobial susceptibility testing was performed using a MicroScan WalkAway system (Beckman Coulter K.K., Tokyo, Japan). Antimicrobial susceptibility was defined according to the Clinical and Laboratory Standards Institute M100, 28th ed (46).

### Detection of extended-spectrumβ-lactamase genes, phylogenetic classification, and multilocus sequence typing.

The phenotypic detection of extended-spectrum β-lactamase (ESBL) production was performed according to the Clinical and Laboratory Standards Institute M100, 28th ed (46). For confirmation of ESBL genotype, PCR analysis was performed, as described previously (47).

Affiliation of E. coli isolates to phylogenetic groups, such as A, B1, B2, C, D, E, and F, was determined by the PCR method, as described previously (6, 48–51). For the rapid identification of E. coli STs 69, 73, 95, and 131, a multilocus sequence typing PCR method (52) was used. Other STs were determined according to the Achtman's seven-locus multilocus sequence typing method. Fragments of seven housekeeping genes (*adk*, *fumC*, *gyrB*, *icd*, *mdh*, *purA*, and *recA*) distributed around the E. coli chromosome were amplified by using primers, as described previously (53). PCR was performed using TaKaRa *Ex Taq* (TaKaRa Bio Inc., Shiga, Japan). The amplified PCR products were sequenced bidirectionally using the Sanger DNA sequencing method at Eurofins Genomics (Ebersberg, Germany), and the sequences were assembled into single contigs using ChromasPro software (version 2.1.8; Technelysium Pty. Ltd, South Brisbane, Australia). The allelic profile for each E. coli isolate was determined, and the STs were assigned using the Enterobase genome database (http://enterobase.warwick.ac.uk/mlst/dbs/Ecoli).

### Virulence factor determination.

*E.coli* isolates were screened for carriage of the following 20 VF genes: *afaB/C* (afimbrial adhesin), *fimH* (type 1 fimbriae), *sfaD/E* (S fimbriae), *papC*, *papG2* (P fimbriae), *iha* (IrgA homologue adhesin), *iucD* (aerobactin), *iutA* (aerobactin), *fyuA* (yersiniabactin), *iroN* (catecholate siderophore), *cnf1* (cytotoxic necrotizing factor 1), *hlyA* (alpha hemolysin), *sat* (secreted autotransporter toxin), *KpsMT2* (protectin), *usp* (uropathogenic specific protein), *ibeA* (invasion of brain endothelium), *traT* (serum/complement resistance), *cvaC* (colicin V), *ompT* (outer membrane protease T), and *TcpC* (Toll/interleukin-1 receptor domain-containing protein C). PCR assays were used to reveal the prevalence of these virulence genes using specific primers, as described previously (54–66). PCR was performed using EmeraldAmp MAX PCR Master Mix (TaKaRa Bio Inc., Shiga, Japan). The primers used in this study are listed in Table S1.

VF scores were defined as the number of VFs detected for each isolate.

### Biofilm formation assay.

The biofilm formation of E. coli strains was investigated in microtiter plates as described previously (67), with minor modifications. Briefly, each E. coli strain was cultured overnight in 10 ml LB Lennox broth or brain heart infusion (BHI) broth at 37°C, and then the bacterial culture was diluted into fresh LB Lennox broth or BHI broth (Becton, Dickinson, and Company, Sparks, MD) and adjusted to the turbidity of a 0.5 McFarland standard (McFarland Densitometer DEN-1B, Wakenbtech Co., Ltd., Kyoto, Japan). For LB Lennox broth, 1 g tryptone, 0.5 g yeast extract, and 0.5 g NaCl were suspended in 100 ml of distilled water. The dilutions were added to a polystyrene 96-well dish (100 μl/well) with six replicate wells for each strain, and the microtiter plate was incubated for 24 h at 37°C. After incubation, each well was washed three times with sterile distilled water and 0.1% crystal violet solution (Wako Pure Chemical Industries, Ltd, Osaka, Japan), and incubated at room temperature for 10 min. The microtiter plate was rinsed three times by submerging in sterile distilled water and drying overnight. Next, the biofilm stain was dissolved using 125 μl of 30% acetic acid (Wako Pure Chemical Industries, Ltd, Osaka, Japan), and the absorbance was quantified using a plate reader (MultiSkan FC, Thermo Fisher Scientific K.K., Tokyo, Japan) at 550 nm. The tested strains were classified according to their ability to form biofilms, as previously suggested (68). The optical density (OD) cutoff value was defined as three standard deviations above the average OD of the negative control. If the average OD of an E. coli strain was larger than the OD cutoff value, the E. coli strain was defined as a biofilm former. The averages are the results of at least three trials.

### Statistical analysis.

Continuous variables were compared using Student's *t* test. Categorical variables were expressed as numbers and percentages and were compared using Fisher’s two-tailed exact test. For multiple comparison tests, *post hoc* analyses were performed with the Bonferroni correction. Statistical significance was set at *P* < 0.05. All statistical analyses were performed using JMP Pro version 14 software (SAS Institute Inc., Cary, NC).
