# Supplementary material for: Clinical and Microbiological Characteristics of Recurrent Escherichia coli Bacteremia
Source: Microbiol Spectr. 2021 Dec 8;9(3):e01399-21. doi: 10.1128/Spectrum.01399-21 (PMC8653835; doi:10.1128/Spectrum.01399-21)
Supplement: SUPPLEMENTAL FILE 1 — Supplemental material. Download SPECTRUM01399-21_Supp_1_seq7.pdf, PDF file, 0.4 MB [file spectrum01399-21_supp_1_seq7.pdf]

Table S1. The Oligonucleotide primers used in this study

PCR method	Primer name	Primer sequences(5'→3')	Reference
ERIC-PCR	ERIC1R	ATGTAAGCTCCTGGGGATTCAC	(1)
	ERIC2	AAGTAAGTGACTGGGGTGAGCG	(1)
detection of ESBL genes	CTX-M-1-F	GCTGTTGTTAGGAAGTGTGC	(2)
	CTX-M-1-R	CCATTGCCCGAGGTGAAG	(2)
	CTX-M-9-F	GCAGATAATACGCAGGTG	(2)
	CTX-M-9-R	CGGCGTGGTGGTGTCTCT	(2)
phylogenetic classification	chuA.1b	ATGGTACCGGACGAACCAAC	(3)
	chuA.2	TGCCGCCAGTACCAAAGACA	(4)
	yjaA.1b	CAAACGTGAAGTGTCAGGAG	(3)
	yjaA.2b	AATGCGTTCCTCAACCTGTG	(3)
	TspE4C2.1b	CACTATTCGTAAGGTCATCC	(3)
	TspE4C2.2b	AGTTTATCGCTGCGGGTCGC	(3)
	AceK.f	AACGCTATTCGCCAGCTTGC	(3)
	ArpA1.r	TCTCCCCATACCGTACGCTA	(5)
	ArpAgpE.f	GATTCCATCTTGTCAAAATATGCC	(6)
	ArpAgpE.r	GAAAAGAAAAAGAATTCCCAAGAG	(6)
	trpAgpC.1	AGTTTTATGCCCAGTGCGAG	(6)
	trpAgpC.2	TCTGCGCCGGTCACGCCC	(6)
	trpBA.f	CGGCGATAAAGACATCTTCAC	(7)
	trpBA.r	GCAACGCGGCCTGGCGGAAG	(7)
MLST-PCR	ST73_for	TGGTTTTACCATTTTGTCGGA	(8)
	ST73_rev	GGAAATCGTTGATGTTGGCT	(8)
	ST131_for	GACTGCATTTCGTCGCCATA	(8)
	ST131_rev	CCGGCGGCATCATAATGAAA	(8)
	ST95_for	ACTAATCAGGATGGCGAGAC	(8)
	ST95_rev	ATCACGCCCATTAATCCAGT	(8)
	ST69_for	ATCTGGAGGCAACAAGCATA	(8)
	ST69_rev	AGAGAAAGGGCGTTCAGAAT	(8)
	adk_F	ATTCTGCTTGGCGCTCCGGG	(9)
	adk_R	CCGTCAACTTTCGCGTATTT	(9)
	fumC_F	TCACAGGTCGCCAGCGCTTC	(9)
	fumC_R	GTACGCAGCGAAAAAGATTC	(9)
	gyrB_F	TCGGCGACACGGATGACGGC	(9)

	gyrB_R	ATCAGGCCTTCACGCGCATC	(9)
	icd_F	ATGGAAAGTAAAGTAGTTGTTCCGGCACA	(9)
	icd_R	GGACGCAGCAGGATCTGTT	(9)
	mdh_F1	AGCGCGTTCTGTTCAAATGC	(9)
	mdh_R1	CAGGTTCAGAACTCTCTCTGT	(9)
	purA_F1	TCGGTAACGGTGTTGTGCTG	(9)
	purA_R	CATACGGTAAGCCACGCAGA	(9)
	recA_F	CGCATTCGCTTTACCCTGACC	(9)
	recA_R	TCGTCGAAATCTACGGACCGGA	(9)
VF determination	afa1	GCTGGGCAGCAAACTGATAATCTC	(10)
	afa2	CATCAAGCTGTTTGTTCGTCCGCCG	(10)
	fimH_F	TGCAGAACGGATAAGCCGTGG	(11)
	fimH_R	GCAGTCACCTGCCCTCCGGTA	(11)
	sfa1	CTCCGGAGAACTGGGTGCATCTTAC	(10)
	sfa2	CGGAGGAGTAATTACAAACCTGGCA	(10)
	pap1	GACGGCTGTACTGCAGGGTGTGGCG	(10)
	pap2	ATATCCTTTCTGCAGGGATGCAATA	(10)
	papG2_F	GGGATGAGCGGGCCTTTGAT	(12)
	papG2_R	CGGGCCCCCAAGTAACTCG	(12)
	iha_F	CTGGCGGAGGCTCTGAGATCA	(13)
	iha_R	TCCTTAAGCTCCCGCGGCTGA	(13)
	Aer1	TACCGGATTGTCATATGCAGACCGT	(14)
	Aer2	AATATCTTCCTCCAGTCCGGAGAAG	(14)
	AerJ_F	GGCTGGACATCATGGGAACTGG	(15)
	AerJ_R	CGTCGGGAACGGGTAGAATCG	(15)
	fyuA_F	TGATTAACCCCGCGACGGGAA	(11)
	fyuA_R	CGCAGTAGGCACGATGTTGTA	(16)
	iroN_F	AAGTCAAAGCAGGGGTTGCCCG	(17)
	iroN_R	GACGCCGACATTAAGACGCAG	(17)
	cnf1	AAGATGGAGTTICCTA’IGCAGGAG	(14)
	cnf2	CATTCAGAGTCCTGCCCTCATTATT	(14)
	sat_F	GTTGTCTCTGGCTGTTGC	(18)
	sat_R	AATGATGTTCCTCCAGAGC	(18)
	hly_F	AACAAGGATAAGCACTGTTCTGGCT	(14)
	hly_R	ACCATATAAGCGGTCATTCCCGTCA	(14)

kpsMTII_F	GCGCATTTGCTGATACTGTTG	(11)
kpsMTII_R	CATCCAGACGATAAGCATGAGCA	(11)
usp_N6	ATGCTACTGTTTCCGGGTAGTGTGT	(19)
usp_N7	CATCATGTAGTCGGGGCGTAACAAT	(19)
ibe10_F	AGGCAGGTGTGCGCCGCGTAC	(20)
ibe10_R	TGGTGCTCCGGCAAACCATGC	(11)
TraT_F	GGTGTGGTGCGATGAGCACAG	(11)
TraT_R	CACGGTTCAGCCATCCCTGAG	(11)
ColV-C_F	CACACACAAACGGGAGCTGTT	(11)
ColV-C_R	CTTCCCGCAGCATAGTTCCAT	(11)
ompT_F	TCATCCCGGAAGCCTCCCTCACTACTAT	(21)
ompT_R	TAGCGTTTGCTGCACTGGCTTCTGATAC	(21)
TcpC_F	GGCAACAATATGTATAATATCCT	(22)
TcpC_R	GCCCAGTCTATTTCTGCTAAAGA	(22)

ERIC-PCR: enterobacterial repetitive intergenic consensus polymerase chain reaction, ESBL: extended-spectrum β-lactamase, VF: virulence factor, MLST: multilocus sequence typing

Table S2: Distribution of STs, phylogenetic group, biofilm formation ability, and possession of ESBL gene of each genetically identical *E. coli* strain

Isolate Number	ST	phylogenetic group	biofilm formation(LB)	biofilm formation(BHI)	possession of ESBL gene
1	ST457	F	-	-	-
2	ST569	B2	-	-	-
3	ST362	E	-	-	-
4	ST1193	B2	+	-	-
5	ST73	B2	+	-	-
6	ST131	B2	-	+	CTX M-9
7	ST95	B2	+	-	-
8	ST83	B2	-	-	-
9	ST95	B2	-	-	-
10	ST131	B2	+	-	CTX M-1
11	ST73	B2	+	-	-
12	ST357	B2	-	-	-
13	ST95	B2	+	-	-
14	ST95	B2	+	-	-
15	ST131	B2	+	+	-
16	ST95	B2	-	-	-
17	ST73	B2	-	-	-
18	ST1193	B2	-	-	CTX M-9
19	ST73	B2	-	-	-
20	ST131	B2	-	-	-
21	ST131	B2	-	-	-
22	ST73	B2	+	-	-
23	ST131	B2	-	+	-
24	ST6769	E	-	-	-
25	ST95	B2	+	+	-
26	ST131	B2	-	-	-
27	new ST	B2	+	-	-
28	ST1193	B2	-	-	-
29	ST131	B2	-	-	-
30	ST131	B2	+	+	-
31	ST95	B2	+	-	-
32	ST131	B2	-	-	CTX M-1
33	ST95	B2	+	-	-
34	ST70	B2	-	+	-
35	ST131	B2	+	+	CTX M-9
36	ST131	B2	-	+	CTX M-9
37	ST131	B2	-	-	-
38	ST131	B2	+	+	CTX M-9

39	ST405	E	-	+	CTX M-1
40	ST95	B2	+	+	-
41	ST131	B2	+	+	CTX M-9
42	ST131	B2	+	+	CTX M-9
43	ST95	B2	+	+	-
44	ST69	E	-	-	-
45	ST73	B2	+	+	-
46	ST62	F	-	-	-
47	ST73	B2	-	+	-
48	ST131	B2	-	-	-
49	ST131	B2	-	+	CTX M-9

ST, sequence type, ESBL, extended-spectrum β-lactamase, BHI, brain heart infusion

Table S3: Distribution of STs, phylogenetic group, biofilm formation ability, and possession of ESBL gene of each genetically discordant *E. coli* strain

Isolate Number	ST	phylogenetic group	biofilm formation (LB)	biofilm formation (BHI)	possession of ESBL gene
50	ST357	B2	-	-	-
51	ST88	A	-	+	-
52	ST95	B2	+	+	-
53	new ST	E	-	-	-
54	ST4623	B1	-	-	-
55	ST38	E	+	-	CTX M-9
56	ST453	B1	-	-	-
57	new ST	B1	-	-	-
58	new ST	B2	-	-	-
59	ST1611	B1	-	+	-
60	ST1193	B2	-	-	-
61	ST648	F	-	-	-
62	ST538	B2	+	+	CTX M-9
63	ST117	F	-	+	-
64	ST131	B2	-	+	-
65	ST162	B1	-	-	-
66	ST95	B2	+	-	-
67	ST131	B2	-	-	-
68	ST131	B2	+	+	-
69	ST297	B1	-	-	-
70	ST95	B2	+	+	-

ST, sequence type, ESBL, extended-spectrum β-lactamase, BHI, brain heart infusion

Table S4: Distribution of 20 virulence factor genes and VF scores of each genetically identical *E. coli* strain

Isolate Number	*afaB/C*	*iucD*	*CNF1*	*sfaD/E*	*papC*	*fyuA*	*cvaC*	*fimH*	*iutA*	*ibeA*	*iha*	*ompT*	*KpsMT2*	*papG2*	*hlyA*	*TcpC*	*usp*	*iroN*	*sat*	*traT*	VF score
1	-	+	-	-	+	-	-	+	+	-	-	-	+	-	-	-	-	-	-	+	6
2	-	-	-	-	-	+	-	+	-	+	-	-	+	-	-	-	+	-	-	-	5
3	-	-	-	-	+	-	-	+	-	-	-	+	-	+	-	-	-	+	-	+	6
4	-	+	-	-	-	+	-	+	+	-	+	-	+	-	-	-	+	-	+	-	8
5	-	+	+	+	+	+	-	+	+	-	+	-	-	+	+	+	+	+	+	+	15
6	-	+	-	-	-	+	-	+	+	-	+	-	+	-	-	-	+	-	+	-	8
7	-	-	-	-	+	+	-	+	-	-	-	-	+	+	-	+	+	-	-	+	8
8	-	-	+	+	+	+	+	+	-	+	-	-	+	-	+	-	+	+	-	+	12
9	-	-	-	-	+	+	-	+	-	+	-	-	+	+	-	-	+	-	-	+	8
10	-	+	+	-	+	+	-	+	+	-	+	-	-	+	+	-	+	-	+	+	12
11	-	+	+	+	+	+	-	+	+	-	+	-	+	+	+	+	+	+	+	+	16
12	-	-	-	-	-	+	-	+	-	+	-	-	+	-	-	-	+	-	-	+	6
13	-	-	+	+	+	+	-	+	-	-	-	-	+	-	+	+	+	+	-	+	11
14	-	-	-	-	+	+	-	+	-	+	-	-	+	+	-	-	+	-	-	+	8
15	-	+	-	-	-	+	-	+	+	-	+	-	-	-	-	-	+	-	+	+	8
16	-	-	+	+	+	+	-	+	-	-	-	-	-	-	+	-	+	+	-	-	8
17	-	+	+	-	+	+	-	+	+	-	+	-	+	+	+	+	+	-	+	+	14
18	-	+	-	-	-	+	-	+	+	-	+	-	+	-	-	-	+	-	+	-	8
19	-	-	-	-	-	+	-	+	-	+	-	-	+	-	-	-	+	-	-	-	5
20	-	+	-	-	-	+	-	+	+	-	+	-	+	-	-	-	+	-	+	+	9
21	-	+	-	-	-	+	-	+	+	-	+	-	-	-	-	-	+	-	+	+	8
22	-	+	+	+	+	+	-	+	+	-	+	-	+	+	+	+	+	+	+	+	16
23	+	+	-	-	-	+	-	+	+	-	+	-	+	-	-	-	+	-	+	+	10
24	-	+	-	-	-	-	-	+	+	-	-	-	+	-	-	-	-	-	-	+	5
25	-	-	-	-	+	+	+	+	-	-	-	+	+	+	-	-	+	+	-	+	10
26	-	+	-	-	-	+	-	+	+	-	+	-	+	-	-	-	+	-	+	-	8

27	-	-	-	-	-	+	-	+	-	+	-	-	+	-	-	-	+	+	-	+	7
28	-	+	-	-	-	+	-	+	+	-	+	-	+	-	-	-	+	-	+	-	8
29	-	+	-	-	-	+	-	+	+	-	+	-	+	-	-	-	+	-	+	-	8
30	-	-	-	-	-	+	-	+	-	-	+	-	-	-	-	-	+	-	-	+	5
31	-	-	-	-	+	+	-	+	-	-	-	-	+	+	-	+	+	-	-	+	8
32	+	+	-	-	-	+	-	+	+	-	+	-	+	-	-	-	+	-	+	+	10
33	-	-	-	-	+	+	-	+	-	+	-	-	+	+	-	-	+	-	-	+	7
34	-	-	-	-	-	+	-	+	-	-	-	-	-	-	-	-	-	-	-	+	3
35	-	+	-	-	-	+	-	+	+	-	+	-	+	-	-	-	+	-	+	+	9
36	-	+	-	-	-	+	-	+	+	-	+	-	+	-	-	-	+	-	+	+	9
37	+	+	-	-	-	+	-	+	+	+	+	-	+	-	-	-	+	-	+	+	11
38	-	+	-	-	-	+	-	+	+	-	+	-	-	-	-	-	+	-	+	+	8
39	-	+	-	-	-	+	-	+	+	-	-	-	+	-	-	-	-	-	-	+	6
40	-	-	+	+	+	+	-	+	-	+	-	-	+	-	+	-	+	+	-	+	11
41	-	+	-	-	+	+	-	+	+	-	+	-	+	-	-	-	+	-	+	+	10
42	-	+	-	-	-	+	-	+	+	-	+	-	+	-	-	-	+	-	+	+	9
43	-	-	+	-	+	+	-	+	-	-	-	-	-	-	+	-	+	-	-	+	7
44	-	+	-	-	-	+	+	+	+	-	-	+	+	-	-	-	-	+	-	+	9
45	-	-	+	+	+	+	-	+	-	-	-	-	+	+	+	-	+	+	-	-	10
46	-	+	-	-	+	+	-	+	+	-	+	-	+	+	-	-	-	-	+	+	10
47	-	+	-	+	-	+	-	+	+	-	+	-	+	-	-	+	+	+	+	+	12
48	-	+	-	-	-	+	-	+	+	-	+	-	+	-	-	-	+	-	+	+	9
49	-	-	-	-	-	+	-	+	-	-	-	-	-	-	-	-	+	-	-	+	4

Table S5: Distribution of 20 virulence factor genes and VF scores of each genetically discordant *E. coli* strain

Isolate Number	*afaB/C*	*iucD*	*CNF1*	*sfaD/E*	*papC*	*fyuA*	*cvaC*	*fimH*	*iutA*	*ibeA*	*iha*	*ompT*	*KpsMT2*	*papG2*	*hlyA*	*TcpC*	*usp*	*iroN*	*sat*	*traT*	VF score
50	-	-	-	-	-	+	-	+	-	+	-	-	+	-	-	-	+	-	-	+	6
51	-	+	-	-	-	+	+	+	+	-	-	+	-	-	-	-	-	+	-	+	8
52	-	-	-	-	+	+	-	+	-	-	-	-	+	+	-	-	+	-	-	+	7
53	-	-	-	-	-	-	-	+	-	-	-	-	+	-	-	-	-	-	-	-	2
54	-	-	-	-	-	-	-	+	-	-	-	-	-	-	-	-	-	-	-	-	1
55	+	-	-	-	-	+	-	-	-	-	-	-	+	-	-	-	-	-	-	+	4
56	-	-	-	-	-	+	-	+	-	-	-	-	+	-	-	-	+	-	-	+	5
57	-	-	-	-	-	+	-	+	-	-	-	-	-	-	-	-	-	-	-	-	2
58	-	-	-	-	-	-	-	+	-	-	-	-	-	-	-	-	-	-	-	-	1
59	-	-	-	-	-	-	-	+	-	-	-	-	-	-	-	-	-	-	-	-	1
60	-	+	-	-	-	+	-	+	+	-	+	-	+	-	-	-	+	-	+	-	8
61	-	+	-	-	-	-	+	+	+	-	-	+	+	-	-	-	-	+	-	+	8
62	-	-	-	-	-	+	-	+	-	+	-	-	-	-	-	-	+	-	-	-	4
63	-	+	-	-	-	-	+	+	+	-	-	+	-	-	-	-	-	+	-	+	7
64	-	+	-	-	-	+	-	+	+	-	+	-	+	-	-	-	+	-	+	+	9
65	-	+	-	-	-	-	+	+	+	-	-	+	-	-	-	-	-	+	-	+	7
66	-	-	-	-	+	+	-	+	-	-	-	-	+	+	-	+	+	-	-	+	8
67	-	+	-	-	+	+	-	+	+	+	+	-	+	-	-	-	+	-	+	-	10
68	-	+	-	-	-	+	-	+	+	-	+	-	+	-	-	-	+	-	+	+	9
69	-	-	-	-	-	-	-	+	-	-	-	-	-	-	-	-	-	-	-	-	1
70	-	-	-	-	+	+	-	+	-	-	-	-	+	+	-	+	+	-	-	+	8

VF, virulence factor

References

1. Versalovic J, Koeuth T, Lupski JR. 1991. Distribution of repetitive DNA sequences in eubacteria and application to fingerprinting of bacterial genomes. Nucleic Acids Res 19:6823-31.
2. Shibata N, Kurokawa H, Doi Y, Yagi T, Yamane K, Wachino J, Suzuki S, Kimura K, Ishikawa S, Kato H, Ozawa Y, Shibayama K, Kai K, Konda T, Arakawa Y. 2006. PCR classification of CTX-M-type beta-lactamase genes identified in clinically isolated gram-negative bacilli in Japan. Antimicrob Agents Chemother 50:791-5.
3. Clermont O, Christenson JK, Denamur E, Gordon DM. 2013. The Clermont Escherichia coli phylo-typing method revisited: improvement of specificity and detection of new phylo-groups. Environ Microbiol Rep 5:58-65.
4. Clermont O, Bonacorsi S, Bingen E. 2000. Rapid and simple determination of the Escherichia coli phylogenetic group. Appl Environ Microbiol 66:4555-8.
5. Clermont O, Bonacorsi S, Bingen E. 2004. Characterization of an anonymous molecular marker strongly linked to Escherichia coli strains causing neonatal meningitis. J Clin Microbiol 42:1770-2.
6. Lescat M, Clermont O, Woerther PL, Glodt J, Dion S, Skurnik D, Djossou F, Dupont C, Perroz G, Picard B, Catzeflis F, Andremont A, Denamur E. 2013. Commensal Escherichia coli strains in Guiana reveal a high genetic diversity with host-dependant population structure. Environ Microbiol Rep 5:49-57.
7. Clermont O, Lescat M, O'Brien CL, Gordon DM, Tenaillon O, Denamur E. 2008. Evidence for a human-specific Escherichia coli clone. Environ Microbiol 10:1000-6.
8. Doumith M, Day M, Ciesielczuk H, Hope R, Underwood A, Reynolds R, Wain J, Livermore DM, Woodford N. 2015. Rapid identification of major Escherichia coli sequence types causing urinary tract and bloodstream infections. J Clin Microbiol 53:160-6.
9. Wirth T, Falush D, Lan R, Colles F, Mensa P, Wieler LH, Karch H, Reeves PR, Maiden MC, Ochman H, Achtman M. 2006. Sex and virulence in Escherichia coli: an evolutionary perspective. Mol Microbiol 60:1136-51.
10. Le Bouguenec C, Archambaud M, Labigne A. 1992. Rapid and specific detection of the pap, afa, and sfa adhesin-encoding operons in uropathogenic Escherichia coli strains by polymerase chain reaction. J Clin Microbiol 30:1189-93.
11. Johnson JR, Stell AL. 2000. Extended virulence genotypes of Escherichia coli strains from patients with urosepsis in relation to phylogeny and host compromise. J Infect Dis 181:261-72.
12. Johnson JR, Brown JJ. 1996. A novel multiply primed polymerase chain reaction assay

for identification of variant papG genes encoding the Gal(alpha 1-4)Gal-binding PapG adhesins of Escherichia coli. J Infect Dis 173:920-6.

13. Johnson JR, Russo TA, Tarr PI, Carlino U, Bilge SS, Vary JC, Jr., Stell AL. 2000. Molecular epidemiological and phylogenetic associations of two novel putative virulence genes, iha and iroN(E. coli), among Escherichia coli isolates from patients with urosepsis. Infect Immun 68:3040-7.
14. Yamamoto S, Terai A, Yuri K, Kurazono H, Takeda Y, Yoshida O. 1995. Detection of urovirulence factors in Escherichia coli by multiplex polymerase chain reaction. FEMS Immunol Med Microbiol 12:85-90.
15. Johnson JR, Stapleton AE, Russo TA, Scheutz F, Brown JJ, Maslow JN. 1997. Characteristics and prevalence within serogroup O4 of a J96-like clonal group of uropathogenic Escherichia coli O4:H5 containing the class I and class III alleles of papG. Infect Immun 65:2153-9.
16. Schubert S, Rakin A, Karch H, Carniel E, Heesemann J. 1998. Prevalence of the "high-pathogenicity island" of Yersinia species among Escherichia coli strains that are pathogenic to humans. Infect Immun 66:480-5.
17. Rodriguez-Siek KE, Giddings CW, Doetkott C, Johnson TJ, Fakhr MK, Nolan LK. 2005. Comparison of Escherichia coli isolates implicated in human urinary tract infection and avian colibacillosis. Microbiology (Reading) 151:2097-2110.
18. Ananias M, Yano T. 2008. Serogroups and virulence genotypes of Escherichia coli isolated from patients with sepsis. Braz J Med Biol Res 41:877-83.
19. Nakano M, Yamamoto S, Terai A, Ogawa O, Makino SI, Hayashi H, Nair GB, Kurazono H. 2001. Structural and sequence diversity of the pathogenicity island of uropathogenic Escherichia coli which encodes the USP protein. FEMS Microbiol Lett 205:71-6.
20. Huang SH, Wass C, Fu Q, Prasadarao NV, Stins M, Kim KS. 1995. Escherichia coli invasion of brain microvascular endothelial cells in vitro and in vivo: molecular cloning and characterization of invasion gene ibe10. Infect Immun 63:4470-5.
21. Johnson TJ, Wannemuehler Y, Doetkott C, Johnson SJ, Rosenberger SC, Nolan LK. 2008. Identification of minimal predictors of avian pathogenic Escherichia coli virulence for use as a rapid diagnostic tool. J Clin Microbiol 46:3987-96.
22. Cirl C, Wieser A, Yadav M, Duerr S, Schubert S, Fischer H, Stappert D, Wantia N, Rodriguez N, Wagner H, Svanborg C, Miethke T. 2008. Subversion of Toll-like receptor signaling by a unique family of bacterial Toll/interleukin-1 receptor domain-containing proteins. Nat Med 14:399-406.
